# Predictive effects of body mass index on immune reconstitution among HIV-infected HAART users in China

**DOI:** 10.1186/s12879-019-3991-6

**Published:** 2019-05-02

**Authors:** Xiaolin Li, Haibo Ding, Wenqing Geng, Jing Liu, Yongjun Jiang, Junjie Xu, Zining Zhang, Hong Shang

**Affiliations:** 1 NHC Key Laboratory of AIDS Immunology (China Medical University), Department of Laboratory Medicine, The First Affiliated Hospital of China Medical University, No 155, Nanjing North Street, Heping District, Shenyang, 110001 Liaoning Province China; 2 Key Laboratory of AIDS Immunology of Liaoning Province, The First Affiliated Hospital of China Medical University, Shenyang, 110001 China; 3 Key Laboratory of AIDS Immunology, Chinese Academy of Medical Sciences, Shenyang, 110001 China; 4 Collaborative Innovation Center for Diagnosis and Treatment of Infectious Diseases, 79 Qingchun Street, Hangzhou, 310003 China

**Keywords:** HIV, Antiretroviral therapy, Body mass index, China, Immune reconstitution

## Abstract

**Background:**

Body mass index (BMI) may contribute somewhat to drug metabolism, and thus affecting the efficacy of highly active antiretroviral therapy (HAART). This study aimed to determine the frequencies of underweight, normal and overweight/obesity at pre-HAART in a large cohort of HIV-infected Chinese patients, and investigate the prospective effects of BMI on immune reconstitution after HAART initiation.

**Methods:**

A longitudinal cohort study was performed to analyze the effects of BMI on immune reconstitution in HIV-infected patients treated with HAART. Multiple linear regression was used to evaluate the relationship between baseline BMI and increased CD4+ T lymphocyte levels at 12 and 30 months after initiating HAART. In addition, Cox proportional hazard model was used to assess the relationship between BMI and time to achieve immunologic reconstitution (CD4+ T lymphocytes>500cells/μL) during the follow-up period.

**Results:**

Among the 1612 enrolled patients, 283 (17.6%) were overweight/obese (BMI ≥ 25 kg/m^2^), 173 (10.7%) were underweight (BMI < 18.5 kg/m^2^) and the remaining were normal weight. Prior to HAART initiating, overweight HIV-infected patients were mostly males, older ages, exhibited higher CD4+ T lymphocytes and lower viral loads (*p* < 0.01 for all). Patients with higher baseline BMI had an independently positive effect on 30-month CD4+ T lymphocyte recovery (*p* = 0.028), but not 12-month CD4+ T lymphocyte gain (*p* = 0.104). In addition, a Cox proportional hazard model with baseline BMI as an independent variable indicated that BMI was correlated with an increased likelihood of achieving immunologic reconstitution over time (hazard ratios [HR] 1.03; 95% confidence intervals [CI] 1.01–1.06; *p* = 0.011), after adjusting for baseline age, gender, CD4+ T lymphocytes, CD4/CD8 ratio, viral load and WHO stage.

**Conclusions:**

Higher baseline BMI could predict better immune reconstitution in HIV-infected patients after HAART initiating.

## Background

Body mass index (BMI) is a critical indicator of nutritional status in patients with human immunodeficiency virus (HIV) infection. The World Health Organization (WHO) has recommended the following categories based on BMI values: underweight (< 18.5 kg/m^2^); normal (18.5–24.9 kg/m^2^); overweight (25–29.9 kg/m^2^); obese (30–39.9 kg/m^2^) and morbidly obese (≥40 kg/m^2^). Emaciation is a common condition during the early period of HIV epidemic. However, with the emergence of highly active antiretroviral therapy (HAART), the frequency of overweight/obesity has risen considerably [1, 2]. For instance, a study in an urban cohort of HIV-infected patients has demonstrated that 45% of that population is overweight/obese, which may be more prevalent than wasting [3].

Typically, obesity can increase the risk of numerous deleterious health consequences in general populations, including hypertension, coronary artery disease, myocardial infarction, diabetes and stroke [4, 5]. In the pre-HAART era, obesity has been associated with delayed disease progression among HIV-infected patients [6, 7]. However, the effects of BMI on immune reconstitution after initiating HAART remain inconsistent. Several studies have reported a comparable immune status among HIV-infected patients under normal, overweight, and obese categories, and higher BMI is not correlated with immunological and viral responses to antiretroviral therapy [8]. In contrast, other reports suggest that the immune reconstitution induced by HAART is often greatest among subjects classified as overweight [9, 10], and there is some evidence that a higher BMI is associated with more robust CD4+ T-cell recovery in HAART-treated patients [11]. Besides, a study discovered that following HAART, obese HIV-infected patients acquired fewer CD4+ T lymphocytes compared to normal weight HIV-infected patients, indicating that the potentially adverse immune response is associated with excess weight [6].

Most of the aforementioned studies are conducted in Western countries. Indeed, Wang et al. [12] suggest that Asians possess lower mean BMI but higher body fat percentage than Caucasians of the same age and gender. Nonetheless, the dosages of antiretroviral drugs used for treating our HIV-infected Asian patients were the same as those used by Caucasians. To date, studies on the clinical characteristics and efficacy of antiretroviral therapy among underweight, normal and overweight/obese HIV-infected adult populations in China are relatively limited. Therefore, this study aimed to assess the frequencies of underweight, normal and overweight/obesity at baseline (pre-HAART) in a large cohort of HIV-infected Chinese patients. In addition, the effects of baseline BMI on HIV prognosis were investigated by measuring CD4+ T lymphocyte levels during routine medical care after HAART initiation.

## Methods

### HIV subjects

A retrospective observational cohort study was conducted on adult patients enrolling in care at the HIV Clinic of the 1st Affiliated Hospital of China Medical University, Shenyang. Patients with HIV-seropositive test results were eligible for this study. The study cohort included HAART-naïve patients who initiated HAART between 2004 and 2016, and underwent follow-up examinations for up to 120 months during antiretroviral therapy. Written informed consent was obtained from all participants prior to enrollment.

### Data collection

At initial clinic visit before treatment, a detailed questionnaire was specifically designed to collect the demographic, clinical and socioeconomic data from all participants, including height, weight, age at HAART initiation, gender, clinical symptoms, history of AIDS-defining events and drug combinations. Other recorded variables were included race (Han and non-Han), alcohol use, tobacco smoking, transmission route (homosexual transmission, heterosexual transmission, injection drug use [IDU], transfusion or other), marital status (single, married/partnered, divorced/separated or widowed) and educational background.

In addition, all patients underwent a complete physical examination and laboratory testing such as cytometry, fasting blood lipids, CD4+ T lymphocytes, HIV-RNA viral load, and co-infection with tuberculosis and hepatitis B/C virus. According to the WHO clinical staging system for HIV/AIDS, the patients were classified as Stage I to IV, depending on their specific clinical conditions and symptoms that reflect disease progression from primary HIV infection to advanced AIDS. Stage IV was characterized by severe opportunistic infection and/or a CD4+ T lymphocyte count of less than 200cells/μL.

After initiating antiretroviral therapy, the patients attended follow-up visits every 3 months during the first year after starting HAART, and then the frequencies of follow-up were reduced to 6-month intervals. Biological specimens were collected during each follow-up visit for routine examination, immunological and viral assays, in order to evaluate adverse events (AEs) and therapeutic effects. All data obtained from patients’ medical records were entered into an electronic database.

### Measurements of BMI and HIV-RNA

The weight of all patients was objectively measured in the morning after fasting for more than 6 h. BMI was calculated by dividing weight in kilograms by height in meters squared (kg/m^2^), and categorized according to above-mentioned WHO guidelines.

An internationally accredited Roche TaqMan viral load method was used to measure and quantify HIV-RNA, which exhibited excellent stability, high sensitivity and low detection limit (20copies/mL). At present, the most commonly used definition of virologic suppression is less than 400copies/mL in most areas of China.

### Statistical analysis

Wilcoxon rank sum test (continuous variables) or χ2 test (discrete variables) was used to compare the differences in demographic and clinical characteristics of HIV patients across different BMI categories. The continuous variables were summarized as medians (inter quartile range [IQR]). Logistic regression models were used to identify the factors that contribute to undetectable viral load (HIV-RNA < 400copies/mL) within 3–6 months after initiating HAART. Besides, univariable and multivariable linear regression (enter) were performed to assess the relationship between the baseline variables, including BMI and CD4+ T cell increase at 12 and 30 months. Viral suppression has been hypothesized to have potential effects on the elevated levels of CD4+ T lymphocytes, and thus virologic failure was incorporated into the model. Patients who did not achieve an viral suppression (HIV-RNA < 400copies/mL) within the first 3–6 months after HAART, or with a plasma HIV-RNA level of greater than or equal to 400copies/mL for two successive censor after achieving <400copies/mL were considered as virologic failure. All variables with *p*-value< 0.2 in the univariate model were included into the multivariate analysis.

Cox proportional hazard model was used to compare hazard ratios (HR) on time to events of immune reconstitution outcomes, with BMI as a continuous variable. The primary outcome measure was time to first occurrence of achieving more than 500cells/μL CD4+ T lymphocytes during the entire course of this study. The event was documented from the date of initial inclusion to the date of the visit in which the event was recorded. The model was adjusted by age, gender, CD4+ T lymphocytes, HIV-RNA viral load and WHO stage at pre-HAART.

All statistical analyses were performed using SPSS version 18.0 (IBM Corp., Armonk, NY, USA). *P* values of less than 0.05 were considered statistically significant.

## Results

### Cross-sectional analysis: relationship of demographics and clinical characteristics with different BMI categories prior to HAART initiation

This study included 1612 eligible HIV-infected patients, of whom 173 (10.7%) were underweight, 1156 (71.7%) were normal weight, 256 (15.9%) were overweight and only 27 (1.7%) were obese. Obese and overweight patients were combined into a single group because obese cohort contained less than 2% of total patients. Demographic characteristics are presented according to BMI strata. Overweight patients were older than underweight and normal weight patients (*p* < 0.01), with median ages of 29, 32 and 35, respectively. Most of the subjects were male (94.2%), while female patients, comprising 5.8% of the cohort, were more likely to be underweight compared to male patients (*p* = 0.004). However, the marital status, educational levels and history of excessive ethanol and smoking did not remarkably differ across BMI groups. The majority of the participants (86.4%) were Han Chinese, and BMI groups did not differ significantly by ethnic group (*p* = 0.613). The association between hepatitis B/C virus serostatus and BMI categories could not be investigated, due to their low overall prevalence rates (< 5% for both subtypes). Besides, the history of AIDS-defining event and tuberculosis co-infection were most frequent (*p* < 0.01) in the underweight HIV-infected patient group (Table 1). An AIDS-defining event was defined as the occurrence of a serious opportunistic infection, including pneumocystis carinii pneumonia (PCP), Kaposi’s sarcoma, oesophageal candidiasis, cytomegalovirus, wasting syndrome, lymphoma, atypical mycobacteria and various presumptive infections. In addition, the frequency of advanced HIV infection (Stage IV) was significantly higher (*p* < 0.01) in underweight group than in the other two BMI groups.

**Table 1:** Cohort Demographic and Clinical Characteristics stratified by body mass index prior to HAART introduction

	Total (*n* = 1612)	Underweight (*n* = 173)	Normal (*n* = 1156)	Overweight/ obese (*n* = 283)	p
Demographic and Clinical Characteristics					
Age	32 (26–44)	29 (24–39)	32 (26–44)	35 (28–45)	< 0.01
Sex					0.004
Male	1519 (94.2)	155 (89.6)	1102 (95.3)	262 (92.6)	
Female	93 (5.8)	18 (10.4)	54 (4.7)	21 (7.4)	
Marital status					0.075
Single	914 (56.8)	111 (64.2)	662 (57.3)	141 (50.0)	
Married/Partnered	483 (30.0)	45 (26.0)	342 (29.6)	96 (34.0)	
Divorced/Separated	196 (12.2)	14 (8.1)	140 (12.1)	42 (14.9)	
Widowed	17 (1.1)	3 (1.7)	11 (1.0)	3 (1.1)	
Ethnics					0.613
Han	1390 (86.4)	153 (88.4)	991 (85.9)	246 (87.2)	
Not Han	218 (13.6)	20 (11.6)	162 (14.1)	36 (12.8)	
Education					0.255
Less than 9 years	438 (28.9)	51 (31.1)	300 (27.8)	87 (32.5)	
More than 9 years	1075 (71.1)	113 (68.9)	781 (72.2)	181 (67.5)	
Smoking					0.278
NO	1000 (62.7)	112 (65.9)	723 (63.2)	165 (58.9)	
YES	594 (37.3)	58 (34.1)	421 (36.8)	115 (41.1)	
Alcohol use					0.104
NO	843 (53.3)	102 (61.1)	594 (52.3)	147 (52.7)	
YES	738 (46.7)	65 (38.9)	541 (47.7)	132 (47.3)	
Transmission route					0.029
Transfusion	4 (0.2)	0 (0.0)	4 (0.3)	0 (0.0)	
IDU	6 (0.4)	2 (1.2)	2 (0.2)	2 (0.7)	
MSM	1292 (80.1)	134 (77.5)	945 (81.7)	213 (75.3)	
HTS	182 (11.3)	20 (11.6)	128 (11.1)	34 (12.0)	
Unkown	128 (7.9)	17 (9.8)	77 (6.7)	34 (12.0)	
Clinical symptoms					< 0.01
Yes	231 (14.3)	51 (29.5)	157 (13.6)	23 (8.1)	
No	1360 (84.4)	121 (69.9)	980 (84.8)	259 (91.5)	
Unkown	21 (1.3)	1 (0.6)	19 (1.6)	1 (0.4)	
Tuberculosis infection					< 0.01
Yes	95 (5.9)	26 (15.0)	62 (5.4)	7 (2.5)	
No	1493 (92.7)	142 (82.1)	1077 (93.2)	274 (96.8)	
Unkown	23 (1.4)	5 (2.9)	16 (1.4)	2 (0.7)	
WHO stage					< 0.01
I	1292 (80.2)	109 (63.0)	932 (80.7)	251 (88.7)	
II	66 (4.1)	7 (4.0)	54 (4.7)	5 (1.8)	
III	104 (6.5)	17 (9.8)	73 (6.3)	14 (4.9)	
IV	149 (9.2)	40 (23.1)	96 (8.3)	13 (4.6)	
Laboratory Examination					
Median CD4 (cells/μL)	243 (139–323)	183 (40–283)	243 (144–318)	271 (181–359)	< 0.01
Median CD8 (cells/μL)	881 (618–1224)	738 (509–1058)	890 (625–1233)	929 (677–1287)	< 0.01
Median CD4/CD8 ratio	0.24 (0.15–0.37)	0.19 (0.07–0.35)	0.25 (0.15–0.37)	0.28 (0.18–0.41)	< 0.01
Median viral load (log10copies/mL)	4.69 (4.25–5.09)	4.87 (4.35–5.43) (*n* = 165)	4.66 (4.25–5.09) (*n* = 1107)	4.71 (4.20–4.97) (*n* = 273)	0.002
Median TC (mmol/L)	3.92 (3.45–4.51)	3.80 (3.40–4.35)	3.89 (3.43–4.50)	4.11 (3.61–4.63)	0.002
Median TG (mmol/L)	1.22 (0.84–1.73)	1.03 (0.75–1.45)	1.18 (0.82–1.67)	1.57 (1.09–2.24)	< 0.01
Median Glucose (mmol/L)	5.18 (4.86–5.59)	4.99 (4.69–5.34)	5.17 (4.85–5.54)	5.39 (5.01–5.93)	< 0.01
Median hemoglobin (g/L)	149 (137–158)	141 (122–153)	149 (137–157)	153 (143–162)	< 0.01

*HAART* highly active antiretroviral therapy, *MSM* men who have sex with men, *HTS* heterosexuality, *IDU* injection drug use, *TC* total cholesterol, *TG* triglyceride

The results of laboratory examinations demonstrated that underweight patients exhibited significantly lower levels of nutritional markers, such as haemoglobin (p < 0.01). On the contrary, the levels of total cholesterol, triglyceride and glucose tended to be increased with increasing BMI category (*p* < 0.01 for all). Regarding the baseline laboratory markers of HIV infection, underweight HIV-infected patients were found to have modestly higher viral loads (*p* = 0.002) than normal and overweight/obese groups. Likewise, prior to HAART initiation, the baseline mean CD4+ T lymphocyte counts and CD4/CD8 ratios of overweight/obese groups were significantly higher (p < 0.01) compared to normal and underweight groups, suggesting that the immune status of overweight/obese HIV-infected patients is comparatively better than the other two BMI groups.

### Longitudinal analysis: association of baseline BMI with immune reconstitution after HAART

**Fig. 1:**
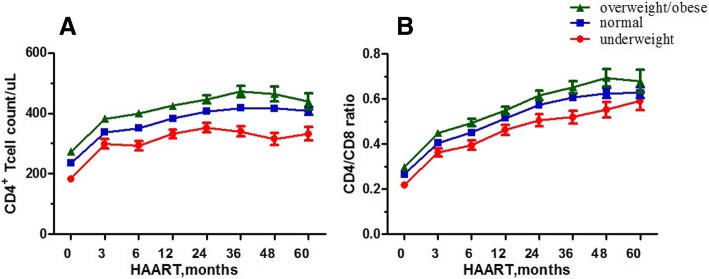
Mean CD4+ T lymphocytes and CD4/CD8 ratio over time on HAART stratified by body mass index

Following HAART initiation, the relationship between BMI and immune reconstitution was assessed. The median duration and regimen of HAART did not differ significantly among the three BMI groups (*p* = 0.945 and *p* = 0.122, respectively). Only a small number of patients were followed for more than 5 years. Beyond that, the CD4+ T lymphocytes and CD4/CD8 ratio of HIV-infected subjects often continued to increase during the first 5-year period after HAART initiation, and the fastest rise in these values occurred within the first 12 months. In addition, during the first 5-year period after treatment, the mean CD4+ T lymphocytes and CD4/CD8 ratio differed among the three BMI groups, the greater values of BMI appeared persistently related to higher mean CD4+ T lymphocyte levels and CD4/CD8 ratios (Fig.1). The data of CD4+ T lymphocytes at 12 and 30 months were available for 1454 (90.2%) and 830 (51.5%) patients, respectively. Notably, CD4+ T cell counts at 12 and 30 months had increased from baseline in 1337 and 784 patients, respectively. Multiple linear regression analysis was performed to evaluate the independent factors associated with increased CD4+ T lymphocyte at both 12 and 30 months. The results showed that baseline BMI was independently predictive of 30-month CD4+ T lymphocyte gain (β = 3.43; 95% confidence intervals [CI] 0.36–6.49; *p* = 0.028) after adjusting for age, gender, baseline CD4+ T lymphocyte count, CD4/CD8 ratio, and WHO stage (Table 2). However, BMI was not significantly correlated with 12-month CD4+ T lymphocyte increase (*p* = 0.104).

**Table 2:** Univariable and multivariable linear regression model of CD4+ T lymphocytes recovery at different time points after antiretroviral therapy initiation

	12-month Increase in CD4 Cell Count				30-month Increase in CD4 Cell Count			
	Univariate Model		Multivariate Model		Univariate Model		Multivariate Model	
Covariate	95% CI	p	95% CI	p	95% CI	p	95% CI	p
Baseline BMI (per 1 kg/m^2^ increase)	1.81 (− 0.11 to 3.74)	0.065	1.64 (−0.34 to 3.61)	0.104	2.29 (−0.67 to 5.25)	0.129	3.43 (0.36 to 6.49)	0.028
Sex	−7.35 (−32.68 to 17.99)	0.570	–	–	49.26 (10.11 to 88.41)	0.014	54.40 (15.24 to 93.55)	0.007
Age	−0.57 (−1.08 to − 0.06)	0.028	–	–	− 0.71 (− 1.47 to − 0.04)	0.065	−0.96 (− 1.74 to − 0.17)	0.017
Drinking	−3.02 (−15.18 to 9.15)	0.627	–	–	8.68 (−9.86 to 27.21)	0.358	–	–
Not Han race	2.68 (−14.62 to 19.97)	0.761	–	–	−5.01 (−33.15 to 23.13)	0.727	–	–
Baseline CD4 (per 100cells/μL increase)	13.70 (9.05 to 18.36)	< 0.01	10.37 (3.82 to 16.93)	0.002	8.42 (0.44 to 16.40)	0.039	–	–
CD4/CD8 ratio	95.06 (59.51 to 130.61)	< 0.01	69.07 (22.29 to 115.84)	0.004	70.63 (12.78 to 128.48)	0.017	69.48 (−1.65 to 140.61)	0.056
Baseline log10 HIV-1 RNA	11.55 (2.96 to 20.14)	0.008	29.213 (19.87 to 38.40)	< 0.01	7.53 (−6.02 to 21.09)	0.275	–	–
Virologic failure	−64.27 (−93.11 to −35.43)	< 0.01	−50.71 (−81.05 to −20.37)	0.001	− 9.18 (−45.28 to 26.91)	0.618	–	–
WHO stage	−8.94 (−15.18 to −2.70)	0.005	–	–	8.67 (−0.54 to 17.89)	0.065	18.51 (8.22 to 28.80)	< 0.01

*CI* confidence intervals

During the follow-up period, among 1561 patients with <500cells/μL CD4+ T lymphocytes at pre-treatment, 750 (48.0%) met the criteria for the primary end point of CD4+ T cell reconstitution during follow-up. A Cox proportional hazard model with continuous BMI as an independent covariate revealed that higher BMI was associated with an increased likelihood of achieving immunologic reconstitution (CD4 > 500cells/μL) after adjusting for age, gender, baseline CD4+ T lymphocytes, CD4/CD8 ratio, viral load and WHO stage (HR = 1.03; 95% CI 1.01–1.06; *p* = 0.011) (Table 3). Furthermore, an adjusted logistic regression model with significant demographic factors indicated that baseline viral load (*p* < 0.01) and CD4+ T lymphocytes (p < 0.01) were the only baseline variables associated with achieving virus suppression within 3–6 months. (Data not shown).

**Table 3:** Effect of BMI on immune reconstitution outcome of CD4 more than 500cells/μL during the follow-up after antiretroviral therapy

Covariate	HR (95% CI)	p	Adjusted HR (95% CI)	p
Continuous BMI	1.06 (1.03 to 1.08)	< 0.01	1.03 (1.01 to 1.06)	0.011
Sex (female vs male)	0.78 (0.56 to 1.07)	0.118	–	–
Age	0.98 (0.97 to 0.98)	< 0.01	0.99 (0.98 to 1.00)	0.004
Baseline CD4 (per 100cells/μL increase)				
1–100	Ref		Ref	
101–200	3.03 (1.95 to 4.70)	< 0.01	2.92 (1.78 to 4.78)	< 0.01
201–300	10.36 (6.91 to 15.52)	< 0.01	10.38 (6.40 to 16.82)	< 0.01
301–400	25.82 (17.12 to 38.93)	< 0.01	25.42 (15.48 to 41.75)	< 0.01
401–499	56.37 (36.40 to 87.30)	< 0.01	52.06 (30.75 to 88.13)	< 0.01
CD4/CD8 ratio	16.07 (11.67 to 22.12)	< 0.01	2.47 (1.47 to 4.13)	0.001
Baseline viral load (per log10 increase) (copies/mL)				
≤ 3.00	Ref		Ref	
≥ 6.01	0.53 (0.23 to 1.25)	0.147	4.60 (1.90 to 11.16)	0.001
5.01–6.00	0.83 (0.44 to 1.57)	0.569	2.62 (1.37 to 4.99)	0.004
4.01–5.00	1.22 (0.65 to 2.28)	0.540	1.97 (1.05 to 3.72)	0.035
3.01–4.00	1.53 (0.80 to 2.92)	0.201	1.68 (0.87 to 3.22)	0.120
WHO stage				
I	Ref		Ref	
II	0.41 (0.27 to 0.63)	< 0.01	0.62 (0.40 to 0.97)	0.037
III	0.34 (0.23 to 0.51)	< 0.01	0.67 (0.44 to 1.04)	0.074
IV	0.22 (0.14 to 0.33)	< 0.01	1.09 (0.68 to 1.75)	0.736
Drinking (yes vs no)	1.04 (0.90 to 1.20)	0.593	–	–
Race (not Han vs Han)	0.98 (0.79 to 1.21)	0.834	–	–

*CI* confidence intervals, *HR* hazard ratios, *Ref* reference

## Discussion

The reconstitution of the immune system is an ultimate goal of HAART, but the host factors contributing to these processes remain largely uncertain. Through a large cohort of HIV-infected adults in China, we found that higher BMI at pre-treatment was associated with advantages in immune recovery over time after HAART initiation. To the best our knowledge, this is the first study to evaluate the impact of BMI on immune reconstitution in a large cohort of Chinese HIV-infected patients.

BMI is a measure of weight relative to height in order to determine body composition. Among the 1612 study participants, the baseline prevalence of overweight/obese was found to be 17.6%. Several recent studies have reported that obesity occurs in 40–60% of HIV-infected adults, whereas our data showed an obviously lower prevalence of overweight among the study participants [9, 13, 14]. These differences may be due to the influence of different dietary patterns on nutritional alterations experienced by patients suffering from HIV infection in each population. Thus, further research is needed to examine the poor nutritional status of HIV-infected patients, such as dietary habits, sleep patterns, employment status and socioeconomic conditions.

Our cross-sectional analyses highlighted significant differences in CD4+ T lymphocytes and HIV-RNA viral load among different BMI groups at baseline (pre-HAART), and the frequency of advanced HIV stage was definitely higher in the underweight group. This can be explained by previous finding that malnutrition is associated with the immune suppression of antigen-specific arms, decreased T-lymphocyte proliferation, and atrophy of lymph tissues. However, most of these studies were conducted on children populations [15–17]. Higher CD4+ T lymphocytes before initiating HAART has been demonstrated to predict better prognosis, and some reports emphasized that patients suffering from pre-treatment immunodeficiency or AIDS-defining conditions may have greater risks of morbidity and mortality both before and during the initial months of HAART [18]. In addition, our results showed that lower baseline HIV-RNA viral loads were associated with achieving virus suppression after 3–6 months of treatment. Thus we propose that higher baseline HIV-1 RNA levels in the underweight strata of our cohort may contribute to the delayed interval before virus inhibition.

Furthermore, higher BMI at pre-HAART was independently related to 30-month increase of CD4+ T lymphocytes and a greater possibility of immune reconstitution (more than 500cells/μL of CD4+ T lymphocytes) during the follow-up period. These findings are somewhat consistent with those reported by Johnson et al [10, 19] that higher BMI predicts greater increases in the amounts of CD4+ T lymphocytes after HAART initiation. To date, the physiological mechanisms linking BMI to the increases of peripheral CD4+ T lymphocytes in HIV-infected patients on HAART remain largely unknown. Several recent studies have shown that obese HIV-negative adults possess higher CD4+ T lymphocytes and CD4/CD8 ratio compared to under or normal weight adults [20, 21]. These phenomena may be explained by the observations that BMI (stratified by obese, overweight and normal) was positively associated with the serum levels of leptin, an adipocyte-derived hormone that influences body weight [22]. Leptin can play an essential role in the immune response to HIV infection by increasing CD4+ T lymphocyte levels, due to the fact that leptin supplementation is associated with CD4+ T lymphocyte proliferation in humans [23, 24]. However, other factors may also affect immunologic reconstitution in patients receiving HAART, such as thymic size and persistent immune activation [25, 26]. Thus, we can surmise that BMI has the potential to play a positive role in controlling immune reconstitution in response to HAART. Moreover, Huang et al. have highlighted that HIV disease progression is more rapid in Chinese men who have sex with men (MSM) than those experienced by MSM in resource-rich settings [27]. Therefore, the strengths of this study not only emphasize that BMI is an easily obtained surveillance indicator predictive of HIV-related health outcomes in some remote areas, but also provide an interesting hypothesis regarding the association between metabolic and immunological cross talks. In the context of HIV-1 infection, metabolism is intimately involved in the regulation and physiology of immune cells, which may represent a mechanistic pathway with therapeutic potential, particularly for underweight patients with poor immune reconstitution in response to HAART. In this study, CD4+ T cell counts significantly rose over a 5-year period after initiating HAART, with the rapidest growth observed during the first year of treatment. These findings were in agreement with some previous reports [28, 29]. In the present study, the baseline BMI was independently related to a greater probability of higher CD4 counts (more than 500cells/μL) and a 30-month increase of CD4+ T lymphocytes, indicating that BMI may predict immune reconstitution in response to HAART after a sufficiently long period of time. In addition, there is evidence suggesting that patients categorized early as having a suboptimal CD4 response may achieve later improvement in CD4 cells over time with continued HAART [30].

The percentage of overweight/obese patients (17.6%) found in this study was comparable to the 17.1% of overweight/obese men and 14.4% of overweight/obese women observed in the general Chinese population. A previous study reported that the obesity rates exhibit an increasing trend from 1993 to 2009 [31]. Thus, it is reasonable to predict that the frequency of overweight/obese HIV-infected patients is continuously increasing, probably due to better access to food and transition to modern sedentary lifestyles. Obesity is associated with an increased risk of diabetes mellitus and cardiovascular disease among HIV-uninfected individuals [32], but each unit increase in BMI may have a more profound effect against the risk of diabetes in HIV-infected patients compared to HIV-uninfected subjects [33, 34]. In contrast, obesity does not appear to adversely affect the key cardiovascular risk factors, such as lipid profile and endothelial activity, in HIV-infected patients [35]. Besides, obesity is associated with both liver steatosis and fibrosis [36], and certain types of cancer [37] in general population. While a recent study indicates that HIV patients receiving HAART with a BMI of approximately 30 kg/m^2^ tend to have a lower risk of developing hepatic, renal, and oncologic diseases compared to normal BMI individuals [38].

Nevertheless, several limitations were inevitable in the present study. First, pharmacokinetic data was not collected, thus, it remained unknown whether the treatment (e.g. drug dosing) of overweight/obese patients could result in drug exposure as compared to normal weight patients. In addition, this observational study might not provide evidence of a direct causal pathway between BMI and immunologic reconstitution. Finally, time-updated BMI was not employed in this study, and therefore the issues of weight gain after HAART initiation had not been considered.

## Conclusions

In summary, our results demonstrate that HIV-infected patients with higher BMI at pre-treatment exhibit better immune reconstitution over time after HAART initiation. Therefore, we should pay more attention to the weight of HIV-infected patients before initiating HAART, in order to predict the immunologic responses to therapy; but that does not imply that overweight patients should be clinically neglected. Future studies are warranted to understand the causal mechanisms underlying the association between BMI, body composition and immune reconstitution in HIV-infected patients, especially via the role of metabolism in regulating immune cell responses.

## Abbreviations

AEs: Adverse events
BMI: Body mass index
CI: Confidence intervals
HAART: Highly active antiretroviral therapy
HIV: Human immunodeficiency virus
HR: Hazard ratios
IDU: Injection drug use
IQR: Inter Quartile Range
MSM: Men who have sex with men
PCP: Pneumocystis Carinii Pneumonia
WHO: World Health Organization
